# Simulating spiking neural networks on massively parallel graphical processing units using a code generation approach with GeNN

**DOI:** 10.1186/1471-2202-15-S1-O1

**Published:** 2014-07-21

**Authors:** Esin Yavuz, James Turner, Thomas Nowotny

**Affiliations:** 1 CCNR, School of Engineering and Informatics, University of Sussex, Falmer, Brighton, BN1 9QJ, UK

## 

A major challenge in computational neuroscience is to achieve high performance for real-time simulations of full size brain networks. Recent advances in GPU technology provide massively parallel, low-cost and efficient hardware that is widely available on the computer market. However, the comparatively low-level programming that is necessary to create an *efficient* GPU-compatible implementation of neuronal network simulations can be challenging, even for otherwise experienced programmers. To resolve this problem a number of tools for simulating spiking neural networks (SNN) on GPUs have been developed [1,2], but using a particular simulator usually comes with restrictions to particular supported neuron models, synapse models or connectivity schemes. Besides being inconvenient, this can unduly influence the path of scientific enquiry.

Here we present GeNN (GPU enhance neuronal networks), which builds on NVIDIA's common unified device architecture (CUDA) to enable a more flexible framework. CUDA allows programmers to write C-like code and execute it on NVIDIA’s massively parallel GPUs. However, in order to achieve good performance, it is critical but not trivial to make the right choices on how to parallelize a computational problem, organize its data in memory and optimize the memory access patterns. GeNN is based on the idea that much of this optimization can be cast into heuristics that allow the GeNN meta-compiler to generate optimized GPU code from a basic description of the neuronal network model in a minimal domain specific language of C function calls. For further simplification, this description may also be obtained by translating variables, dynamical equations and parameters from an external simulator into GeNN input files. We are developing this approach for the Brian 2 [3] and SpineCreator/SpineML [4] systems.

Using a code generation approach in GeNN has important advantages: 1. A large number of different neuron and synapse models can be provided without performance losses in the final simulation code. 2. The generated simulator code can be optimized for the available GPU hardware and for the specific model. 3. The framework is intrinsically extensible: New GPU optimization strategies, including strategies of other simulators, can be added in the generated code for situations where they are effective. The first release version of GeNN is available at http://sourceforge.net/projects/genn. It has been built and optimized for simulating neuronal networks with an anatomical structure (separate neuron populations with sparse or dense connection patterns with the possibility to use some common learning rules).

We have executed performance and scalability tests on an NVIDIA Tesla C2070 GPU with an Intel Xeon(R) E5-2609 CPU running Ubuntu 12.04 LTS. Our results show that as the network size increases, GPU simulations never fail to outperform CPU simulations. But we are also able to demonstrate the performance limits of using GPUs with GeNN under different scenarios of network connectivity, learning rules and simulation parameters, confirming the that GPU acceleration can differ largely depending on the particular details of the model of interest.
